# First Report of Canine Infection by *Leishmania* (*Viannia*) *guyanensis* in the Brazilian Amazon

**DOI:** 10.3390/ijerph17228488

**Published:** 2020-11-16

**Authors:** Francisco J. A. Santos, Luciana C. S. Nascimento, Wellington B. Silva, Luciana P. Oliveira, Walter S. Santos, Délia C. F. Aguiar, Lourdes M. Garcez

**Affiliations:** 1 Seção de Parasitologia, Instituto Evandro Chagas, Secretaria de Vigilância em Saúde, Ministério da Saúde, Ananindeua 67030-000, Pará, Brazil; jralves53@gmail.com (F.J.A.S.); lcassiasn3@gmail.com (L.C.S.N.); luci.oliveiraaa@gmail.com (L.P.O.); waltersantos@iec.gov.br (W.S.S.); 2 Centro de Ciências Biológicas e da Saúde, Universidade do Estado do Pará, Belém 66095-662, Pará, Brazil; 3 Centro Nacional de Primatas, Instituto Evandro Chagas, Ministério da Saúde, Secretaria de Vigilância em Saúde, Ananindeua 67030-000, Pará, Brazil; wellington.silva@cenp.gov.br; 4 Instituto de Ciências Biológicas, Universidade Federal do Pará, Belém 66075-110, Pará, Brazil; delia@ufpa.br

**Keywords:** leishmaniasis, reservoirs, dog, *Leishmania* spp., aetiology, epidemiology

## Abstract

The American cutaneous (CL) and visceral leishmaniasis (VL) are zooanthroponoses transmitted by sand flies. Brazil records thousands of human leishmaniasis cases annually. Dogs are reservoirs of *Leishmania infantum*, which causes VL, but their role in the transmission cycle of CL is debatable. Wild mammals are considered reservoirs of the aetiological agents of CL (*Leishmania* spp.). Objective: To describe the aetiology of leishmaniasis in dogs in an endemic area for CL and VL in the Amazon, Brazil. Methods: Clinical evaluation and blood collection of 40 dogs from the villages Ubim (20) and Socorro (20), city of Tomé-Açu, state of Pará, were carried out. The DNA extracted from the blood was used for PCR with *Leishmania*-specific primers targeting the *hsp70-234* gene sequence. Products were sequenced (ABI3500XL), and the sequences were aligned, edited (BioEdit), and analyzed (Blastn). Results: Of the 34 amplified samples, 21 were sequenced, namely *Leishmania infantum* (12), *L. guyanensis* (5), *L. braziliensis* (3), and *Leishmania* sp. (01). Conclusion: Given the diversity of circulating pathogens, elucidation of the role of the dog in the *Leishmania* spp. cycle in Amazonian villages is imperative to the surveillance of CL in the region. We present the first report in Brazil, confirmed by sequencing, of canine infection by *L. guyanensis*, a species highly resistant to treatment in humans, with the drug of first choice (Glucantime^®^).

## 1. Introduction

Leishmaniasis occurs in more than 90 countries. There are two main clinical forms of the disease, namely visceral leishmaniasis (VL), which compromises internal organs and produces high lethality in untreated cases, and cutaneous leishmaniasis (CL), characterized by skin lesions in most cases (>90%); they may be associated with mucosal involvement or late skin manifestations. The aetiological agents are obligate intracellular protozoa of vertebrates (Kinetoplastida: *Leishmania* spp.). Its incidence is predominant in the poorest regions of the planet [1,2].

The primitive hosts of *Leishmania* spp. are wild mammals of different orders which include Rodentia, Marsupialia, Edentata, Cingulata, Carnivora, Primates, and Chiroptera. Normally, there are no pathological effects of *Leishmania* spp. in wild animals [3]. The wild enzootic cycle of the parasites is maintained between these vertebrates and sand flies (Psychodidae: Phlebotominae). However, synanthropic or domestic wild animals can play an important role as a source of infection to *Leishmania* spp. in the peridomicile (reservoirs), which increases the risk of transmission to humans.

In the Americas, zooanthroponotic cycles of leishmaniasis transmission prevail, where a vector acquires the protozoan by feeding on the blood of animals and then transmits it to humans and domestic animals. In the Amazon region, in Brazil, foxes are incriminated as the main wild reservoirs of *Leishmania infantum* [4], the aetiological agent of VL. However, domestic dogs are the reservoirs with real epidemiological importance in the urban and peri-urban environment, as they are highly susceptible to infection by this species of protozoan and often have high parasitemia and parasitic load on skin tissue [5]. The presence of the vector *Lutzomyia longipalpis*, normally abundant in the home, favors the transmission of VL to people, which occurs mainly in this environment.

In the Amazon, transmission of CL occurs mainly in the forest, with transmission cycles involving sand flies and wild mammals. However, dogs can also become infected and develop skin lesions or an asymptomatic condition. Some of the species causing CL infecting dogs in the Americas are *Leishmania* (*Viannia*) *guyanensis*, *Leishmania* (*Viannia*) *panamensis*, *Leishmania* (*Viannia*) *braziliensis,* and *Leishmania* (*Viannia*) *peruviana* [6,7,8]. Both the species diversity of CL agents and the role of the domestic dog as a reservoir of these *Leishmania* species in the peridomicile remain to be elucidated [9]. In Suriname, it is suspected that the dog is an important link in the chain of transmission of *L.* (*V.*) *guyanensis* to humans [10].

Several molecular markers and molecular techniques are available for leishmaniasis diagnosis by detection, identification, discrimination, and quantification of *Leishmania* parasites. Some of the most used are PCR, RFLP-PCR, and sequencing of *hsp70* and ITS1 markers [11]. The choice of the diagnostic test and the molecular target depends on the objective of the study. In endemic areas with few pathogenic *Leishmania* species circulating, where simple detection or identification of the predominant species is desired, several markers can be successfully used in PCR techniques. An example is a PCR combining the markers SSUr and G6PD to identify *Leishmania* genus, *Leishmania (Viannia)* subgenus, and *L. (V.) braziliensis* species, but lacking the power for discriminating other *Leishmania* species, which are not actually relevant in such an area [12].

Nevertheless, in the Amazon we strongly need to discriminate species since therapy can differ according to the species [13]. The high *Leishmania* intraspecific variability in this region [14] makes it difficult to discriminate species using a PCR (or RFLP-PCR) alone with *hsp70-234*, ITS-1, or other marker, since most of the results for clinical samples are inconclusive or expressed by similar RFLP profiles for different *Leishmania* species [15]. Thus, the sequencing of PCR products cannot be dispensed with to ensure the correct aetiological diagnosis of human or canine leishmaniasis [15].

In Brazil, knowledge about the epidemiological importance of dogs in the transmission chain of human CL is scarce, since the diagnosis of canine infection by *Leishmania* spp., recommended by the Ministry of Health in surveillance actions, does not distinguish between species.

In Tomé-Açu city, Northeast Pará State, the rural villages Socorro and Ubim are the only areas where both clinical forms of human disease are reported. In this Amazonian city, cases of CL associated with infection by *Leishmania* (*Viannia*) *shawi*, *L.* (*V.*) *braziliensis*, and *L.* (*L.*) *infantum* [14] have been described, but no molecular analysis was performed on dog samples living there.

Using DNA extracted from the blood of dogs, we sequenced the *hsp70-234* gene region of *Leishmania* spp. in order to elucidate the aetiology of canine leishmaniasis in the two Amazonian villages of Socorro and Ubim.

## 2. Material and Methods

### 2.1. Characterization of the Tomé-Açu City and the Study Areas

**Figure 1:**
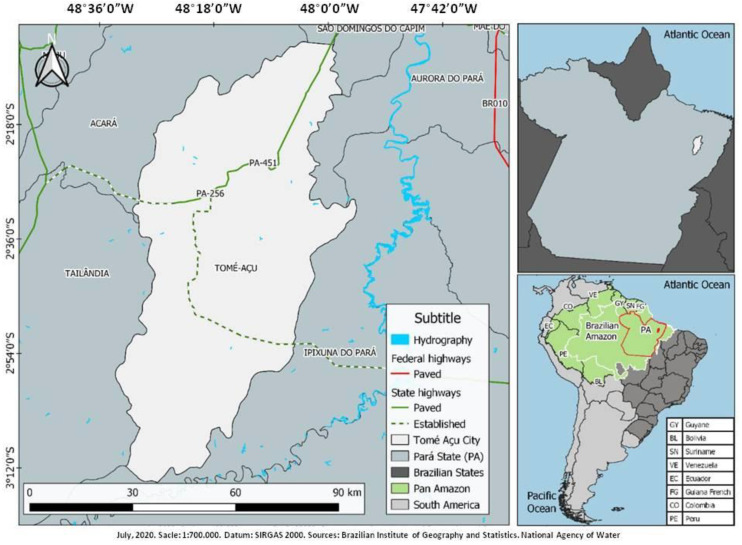
Map of the spatial location of the Amazonian city of Tomé-Açu, state of Pará, Brazil.

Tomé-Açu is an agricultural pole in the northeast of the state of Pará. The Amazonian winter is characterized by heavy rains that occur from December to May, representing 80% of the rains, and the Amazonian summer is the season with the sparsest rainfall, which extends from June to November. The average annual temperature is 26 °C, and the average rainfall is 2300 mm/year. The population in 2019 was estimated at 63,447 inhabitants [16,17]. Figure 1 identifies the location of Tomé-Açu in the northeast of the state of Pará and its distance from the state capital, the city of Belém.

The study areas were the Amazonian villages of Socorro and Ubim, with populations of 1200 and 564 people, respectively, located in the city of Tomé-Açu, state of Pará (Figure 1). They are the only towns in the city where the two forms of the disease coexist. In the other endemic areas of the city of Tomé-Açu, only CL is present.

### 2.2. Dogs, Clinical Examination, and Blood Collection

A series of 40 mixed-breed dogs, resident in the rural villages Socorro (20) and Ubim (20), in May 2019 were analyzed. The animals were examined for the presence of six clinical signs of leishmaniasis, namely alopecia, skin ulcerations, dermatitis, onychogryphosis, conjunctivitis, and lymphadenopathy. Each signal was scored on a semi-quantitative scale from 0 (absent) to 3 (severe). The sum of the values corresponding to the six signs allowed classification of each dog into asymptomatic (0 to 2), oligosymptomatic (3 to 6), or polysymptomatic (7 to 18), as per previously described methods [18]. Then, a sample of peripheral blood (5 mL) from each animal was obtained and stored in a vacuum tube, containing the EDTA anticoagulant (−20 °C), until the moment of analysis. This study was ethically approved according to opinion No. 04/2019 (CEUA-IEC/SVS/MS).

### 2.3. Molecular Methodology

DNA extractions from peripheral blood samples were performed with the Mini Spin 50 Extraction kit (Kasvi^®^, São José dos Pinhais, Brazil), according to the manufacturer’s recommendations, with a final volume of 50 µL.

The samples were analyzed using conventional PCR for the *hsp70-234* target region. The mix, with a final volume equal to 50 µL, contained Taq DNA polymerase 0.03U/µL, 1.5 mM MgCl2, 0.25 mM of each dNTP, 1X buffer solution with KCl, 0.25% DMSO, 0.2 pmol of primers F (5′GGACGAGATCGAGCGCATGGT 3′) and R (5′TCCTTCGACGCCTCCTGGTTG 3′) to amplify the 234 bp [19,20] fragment, and 3.0 µL of DNA from the samples. The test conditions were initial denaturation at 94 °C/5 min, followed by 32 cycles of 94 °C/30 s for denaturation, 61 °C/1 min for annealing, and extension at 72 °C/1 min. The final extension occurred at 72 °C/10 min [19,20]. Two positive controls containing genomic DNA from *L. infantum* (MCER/BR/79/M6445), *L. braziliensis* (MHOM/BR/75/M2904), and a blank control without DNA were included. The amplified DNA samples were subjected to electrophoresis on 2% agarose gel in TAE (Tris acetate 0.004 M; EDTA 0.001 M, pH 8.0) containing GelRed ™ (Biotium^®^, San Francisco, CA, USA) at a concentration of 0.5 μg/mL and, subsequently, observed in a transilluminator for qualitative analysis of the results.

### 2.4. Sequencing

Sequence analysis of the *hsp70-234* gene region was performed for 21 out of 40 samples. For sequencing, the PCR products were purified with the enzyme Illustra ExoProStar 1—Step (GE Healthcare^®^, Buckinghamshire, UK) and prepared for sequencing using the BigDye Terminator Kit v3.1 (Thermo Fisher Scientific^®^, Foster City, CA, USA), following the manufacturer’s specifications. The automatic DNA analyzer model ABI3500XL (Thermo Fisher Scientific^®^) was used to obtain the nucleotide sequences. Each amplicon was sequenced with forward and reverse primers, and the nucleotide sequences were edited and aligned with the aid of the BioEdit [21] program. The final sequences were compared with nucleotide sequences present in GenBank, by observing identity, coverage, e-value, and score, using the BLASTn search tool from NCBI (National Center for Biotechnology Information) to discriminate *Leishmania* species.

## 3. Results

### Clinical Status, Frequency, and Aetiology of Canine Infection

**Figure 2:**
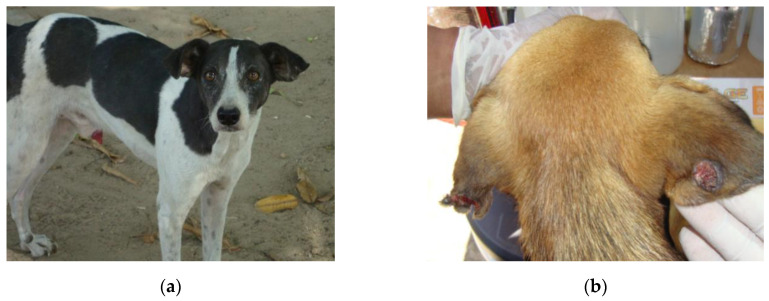
Apparently healthy dog (**a**) and dog with skin lesions (**b**) suggestive of leishmaniasis. Animals from Vila Socorro and Ubim, places where human visceral and cutaneous leishmaniasis coexist, in the Amazon city of Tomé Açu, state of Pará, Brazil (May 2019).

Most dogs did not show signs of canine leishmaniasis (36/40). Among the symptomatic (04/40), three were oligosymptomatic and one was polysymptomatic. Figure 2 depicts an asymptomatic animal (a) and another with skin lesions (b).

**Figure 3:**
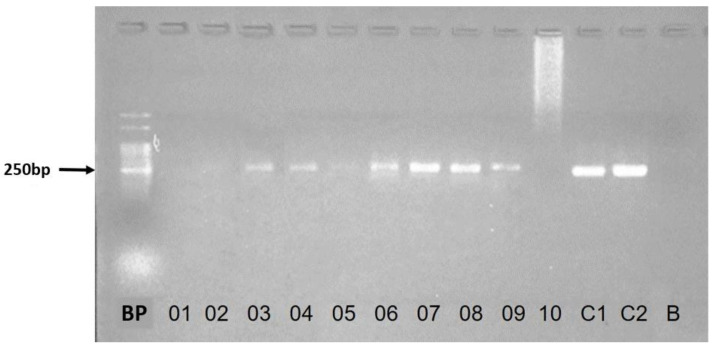
Profile of *hsp70-234* PCR bands visualized on 2% agarose gel. Samples 03, 04, 05, 06, 07, 08, and 09 showed a band pattern close to 250 bp. The standard size of amplicon for this gene region is 234 bp. BP: band pattern 50 bp; C1: positive control, standard strain of *Leishmania* (*Leishmania*) *infantum* (MCER/BR/79/M6445); C2: positive control, standard strain of *Leishmania* (*Viannia*) *braziliensis* (MHOM/BR/75/M2904); B: blank; 01, 02, 03, 04, 05, 06, 07, 08, 09, and 10: blood samples from dogs.

PCR-*hsp70-234* for the detection of the *Leishmania* genus in DNA extracted from blood (Figure 3) revealed an identical frequency of positives (17/20; 85%) in Socorro and Ubim villages. Of the total number of PCR-*hsp70-234* positives (34/40; 95%), the majority (30/34; 88%) were asymptomatic. The sequencing of the *hsp70-234* gene region, performed in 21 of the 34 samples amplified by PCR-*hsp70-234*, allowed discrimination between three species, namely *L.* (*L.*) *infantum* and two other dermotropic species, *L.* (*V.*) *braziliensis* and *L.* (*V.*) *guyanensis* (Table 1). It was not possible to identify the *Leishmania* species in the sample of the polysymptomatic dog, but it was characterized as *Leishmania* (*Leishmania*) sp. (Table 1).

## 4. Discussion

Surveys carried out in zoonotic VL surveillance actions in Brazil allow the diagnosis of *Leishmania* infection in dogs, but without discriminating between species of the parasite. There are few surveys in support of surveillance that address this issue, despite the Ministry of Health of Brazil recommending the discrimination of *Leishmania* species circulating in areas where human VL and CL coexist [17]. In this study, we used molecular methods to elucidate the aetiology of canine *Leishmania* infection in Amazonian villages, endemic to CL and VL. The two villages, Socorro and Ubim, are located in the city of Tomé-Açu, northeast of the state of Pará, in the Brazilian Amazon.

Of the 40 animals included in the study, 36 had no symptoms, but 30 of those asymptomatic (83%) were positive by *hsp70-234* PCR. Subclinical canine leishmaniasis is a common finding in areas with VL transmission in the Amazon [22,23]. Symptomatic dogs are more likely to infect sand fly vectors than asymptomatic ones, but the latter contribute to the maintenance of the *Leishmania* transmission cycle and can also develop high parasitic loads [23]. Therefore, once infected, symptomatic or not, they will be able to transmit the parasite to the sand fly vectors that feed on their blood [24]. These infected vectors may transmit the parasite to humans during a new blood meal.

The period between infection of the dog by a certain species of *Leishmania* and the first clinical manifestations is quite variable. Although it usually lasts for weeks or months, some animals can take several years to become symptomatic [25].

Analysis of DNA extracted from whole-blood samples from 21 of the 34 positive dogs in the two study areas (30 asymptomatic and 4 symptomatic) demonstrated that canine leishmaniasis was associated with at least three species of *Leishmania*, namely *L.* (*L.*) *infantum*, *L.* (*V.*) *braziliensis,* and *L.* (*V.*) *guyanensis*. For the only polysymptomatic dog, the analysis of the *hsp70-234* gene region did not allow discrimination between the species of the parasite, but the subgenus *Leishmania* (*Leishmania*) sp. It could be a variant of *L.* (*L.*) *infantum*, which need to be studied, since there are reports of genetic diversity related to the geographical origin of *L. infantum* isolates in Brazil [26].

In the small series of 21 samples sequenced among the 34 positive, eight animals (38%) were infected with species of the subgenus *Leishmania* (*Viannia*). Although the record of *L.* (*V.*) *guyanensis* in dogs is known in other South American countries, no studies have been found to confirm canine infection by sequencing molecular markers for this species in Brazil. This finding reinforces the importance of using methodologies capable of discriminating between protozoan species for surveillance of LT in areas with great biodiversity such as the Amazon.

In Brazil, the infection of dogs in areas with leishmaniasis transmission is usually attributed to *L. infantum*. The dog, a domestic reservoir of this species, is the target of surveillance and preventive actions for human VL. However, canine infection by *Leishmania* species causing human CL has been increasingly reported in rural villages. Studies indicate that the presence of a dog in the home is a risk factor for the transmission of these pathogens to people [27,28]. In Suriname, the dog may be the domestic reservoir of species of the subgenus *Leishmania* (*Viannia*) [10]. In Colombia, the report of infection in one dog by *L.* (*V.*) *guyanensis* and infection in two others by *L.* (*V.*) *braziliensis*, among 21 positive and sequenced samples (7SL RNA gene), increased the suspicion about its role as reservoir of this pathogen [6]. In both countries, the natural reservoirs of *L.* (*V.*) *guyanensis* are unknown, but in the state of Pará, Brazil, the main reservoirs of *L.* (*V.*) *guyanensis* are Edentates (sloths and anteaters) and several small wild rodents [29].

For humans, infection by *L.* (*V.*) *guyanensis*, very prevalent in Northern Amazonia, represents a high risk of complications, given the parasite’s resistance to the drug of choice for treatment in Brazil (i.e., meglumine antimoniate (Glucantime^®^)) [13]. In Brazilian cities or states where *L.* (*V.*) *guyanensis* predominates, the drug for systemic treatment of the disease should be pentamidine isothyanate, as already occurs in the Amapá state [20].

*L.* (*V.*) *braziliensis* is considered as the main cause of canine cutaneous leishmaniasis and also human cutaneous leishmaniasis in Brazil, given its wide distribution and variety of vectors. In humans, it can cause complications, with extensive or disseminated lesions, which are difficult to resolve, including those in mucous membranes [13].

Leishmaniasis by *L.* (*V.*) *braziliensis* in the dog compromises mainly the skin and, less frequently, can progress to systemic conditions [30]. Some studies on the aetiology of canine infection have been conducted in extra-Amazonian states. In Bahia, a survey in the city of Ilhéus covering 560 dogs confirmed *L.* (*V.*) *braziliensis* in 54.72% of the animals. Most have little or no symptoms [30]. In Pernambuco, 48.7% of the dogs were infected with *L.* (*V.*) *braziliensis* [31]. Other studies with similar results suggest that the clinical course of infection caused by *L.* (*V.*) *braziliensis* is milder compared to that observed in canine infection by *L.* (*L.*) *infantum* [32,33].

Some circumstances may also favor the occurrence of outbreaks of canine leishmaniasis. In Colombia, an outbreak was detected by *L.* (*V.*) *braziliensis* and *L.* (*V.*) *guyanensis* in army animals that entered the forest during training [7].

In the Amazon, potential vectors of pathogens of the subgenus *Leishmania* (*Viannia*) are close to human dwellings, colonizing microenvironments in varying abundance around the home, as we have already observed in the study locations [34]. When dogs living in rural villages accompany their owners to the forest to hunt or to collect food, they are more easily exposed to bites from insects infected with *Leishmania*, which increases the chance of acquiring the infection. Over time, dogs infected with dermotropic species of the parasite may become infective to sand fly vectors. This scenario would increase the risk of transmission of the parasite to humans in the home.

## 5. Conclusions

Given the diversity of pathogens infecting dogs in the study areas, elucidation of the role of the animal in the transmission cycle of *Leishmania* species in Amazonian villages is imperative to the surveillance of CL in the region. Although *L.* (*L.*) *infantum* is the main agent of canine leishmaniasis in the Americas, *L.* (*V.*) *guyanensis* and *L.* (*V.*) *braziliensis* have been associated with 38% of the confirmed canine leishmaniasis cases in Socorro and Ubim villages, where CL and VL coexist. The absence of an association between clinical manifestations and laboratory diagnosis reinforces the indication of the use of molecular tests with discriminatory power for zoonotic leishmaniasis surveillance actions in these areas. This is the first report in Brazil of canine infection by *L.* (*V.*) *guyanensis*, a species of Amazonian distribution highly resistant to treatment in humans with the drug of first choice (Glucantime^®^).

Additionally, this is the first report of *L.* (*V.*) *guyanensis* infecting dogs in Brazil, confirmed by sequencing.

## Figures and Tables

**Table 1:** Canine leishmaniasis: aetiological agents and respective clinical status of dogs in endemic areas for both cutaneous and visceral leishmaniasis. Dogs living in two small Amazonian villages, North Brazil (May 2019).

Villages	Agents	Dogs		Total
		Asymptomatics	Poly/Oligosymptomatics	
Socorro	*L.* (*L.*) *infantum*	5	1	6
	*L.* (*V.*) *guyanensis*	2	0	2
	*L.* (*V.*) *braziliensis*	0	0	0
	*Leishmania* (*L.*) sp.	0	1 ^♣^	1
**Subtotal**		7	2	9
Ubim	*L.* (*L.*) *infantum*	5	1	6
	*L.* (*V.*) *guyanensis*	2	1	3
	*L.* (*V.*) *braziliensis*	3	0	3
**Subtotal**		10	2	12
**Total**		17	4	21

^♣^ Single polysymptomatic dog.
